# Circular economy from scratch: A novel project-based learning method to increase motivation in metal recycling among industrial design students

**DOI:** 10.1016/j.mex.2024.103137

**Published:** 2024-12-27

**Authors:** K. Schoch, M. Bickel, C. Liedtke, F. Hemmert

**Affiliations:** a Faculty of Design and Art, University of Wuppertal, 42119 Wuppertal, Germany; b Division Sustainable Production and Consumption, Wuppertal Institute for Climate, Environment and Energy gGmbH, 42004 Wuppertal, Germany

**Keywords:** Design education, Project-based learning, Motivation, Design for recycling, Industrial design, CE from scratch: A motivating project-based learning method for industrial design students in the context of metal recycling.

## Abstract

Project-based learning, with its emphasis on ‘learning by doing’, is the dominant teaching method in industrial design. Learners are supposed to be motivated to tackle complex problems such as those in the dynamic field of sustainability. However, it is still unclear how the process of increasing motivation within projects can be systematically targeted for specific sustainability challenges and directed towards potential later pro-environmental behavior.

The project-based learning method presented in this paper, framed by a normative decision-making model, aims to intrinsically motivate industrial design students to engage in the exemplary circular economy field of metal recycling and at the same time promote necessary professional competencies on the metal, alloy, product and system level. It is demonstrated which specific intervention measures can be suitable to achieve this goal and how they can be methodically employed. Preliminary quantitative evaluation results indicate that the project-based learning method can indeed strongly motivate the target group.

• Connection to a normative decision-making model from social psychology for the domain of environmental protection as a conceptual framework
• Consideration of intervention measures at various levels (metal, alloy, product, system)
• Use of generative toolkits to translate the acquired knowledge into practical application during design tasks

## Graphical abstract

**Figure:**
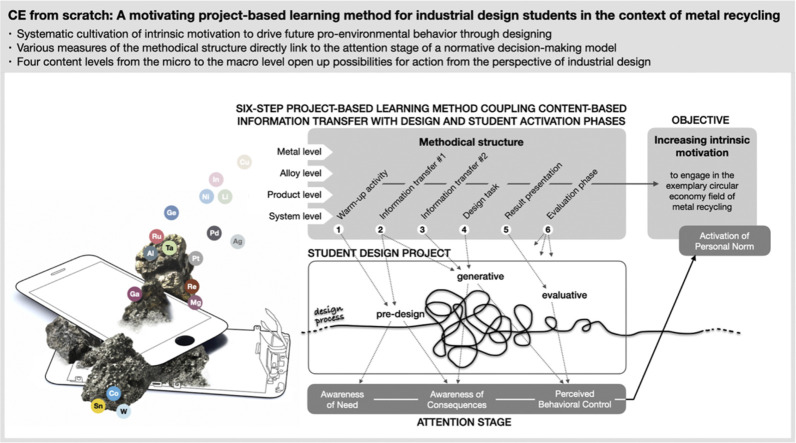

Specifications table

**Table:** 

Subject area:	Environmental Science
More specific subject area:	Design Education
Name of your method:	CE from scratch: A motivating project-based learning method for industrial design students in the context of metal recycling.
Name and reference of original method:	Norm activation models **Matthies, E. (2003). One to bind them all: How the modified moral decision-making model can be used for the integration of measures to promote pro-environmental travel mode choices. In T. Craig (Ed.), *Crossing boundaries—The value of interdisciplinary research* (pp. 103–109). Aberdeen: Robert Gordon University.** Educational perspective **Bell, S. (2010): Project-Based Learning for the 21st Century: Skills for the Future. *The Clearing House*, 83: 39–43.** Practical implementation and speculative design task **Sanders, E.; Stappers, P.J. (2014): Probes, toolkits and prototypes: Three approaches to making in codesigning. *CoDesign*, 10, 1: 5–14.**
Resource availability:	Additional resources will be available upon request.

## Background

Project-based learning is the dominant teaching method in industrial design [1], empowering students to tackle real-world problems and develop essential 21st-century competencies through practical projects [2,3]. A key component of project-based learning is the motivational basis [3,4]. Yet, it remains unclear how to specifically stimulate and systematically guide the process of increasing motivation in industrial design education in such a way that projects promote pro-environmental behavior from scratch. So far, aspects of sustainability in design education are mainly addressed retrospectively through environmental assessment methods, complemented by specific design strategies, or by targeted knowledge transfer [1].

Integrating measures to activate motivation early in product development of projects is crucial because this is when the design brief is most flexible, setting the stage for sustainable products, services or systems [6]. Given the European Union's ongoing push towards a circular economy and the associated regulatory frameworks, such as the Ecodesign for Sustainable Products Regulation [7] and Corporate Sustainability Reporting Directive [8], the demand for a new generation of industrial designers educated in this dynamic field of sustainability continues to grow unabated [9]. Since research on education for the circular economy is still in its early stages [10], there is an even greater need to design target-group specific teaching methods and integrate these into university programs [11,12].

We have developed and evaluated a novel project-based learning method framed by a normative decision-making model in the domain of environmental protection to intrinsically motivate industrial design students to engage in the exemplary circular economy field of metal recycling because, from the European Union's point of view, there is great potential for improvement here [13]. Designing products with recycling in mind can be considered a baseline requirement. Every designed product eventually reaches the end of its lifecycle, and it's essential to preserve its value, whether at the product or material level [14]. In addition to further advantages in ecological and social terms, metal recycling can be particularly relevant for sustaining the functional spectrum of critical metals [15], the European Union heavily relies on [16]. A study of the Fairphone 2 demonstrates that designers play a crucial role in metal recycling by prioritizing modular product structures [17]. Beyond the product level considerations extensively discussed in literature on circular product design [18], strategies to enhance metal recycling can also be found at the material and system levels. The use of low-alloyed materials mitigates the loss of valuable alloying elements and their unique functions into open-loop recycling [19]. Additionally, business model innovations, such as take-back systems and collection services [20], offer promising avenues for increasing the crucial factor of metal collection rates [12].

## Method details

Prior to a detailed description of the project-based learning method, the general process of its derivation is briefly outlined. The design follows the research through design approach, guided by a generic design process [21]. The first three steps of a simple four-step iterative model by Cross [22] are used: At first, the ill-defined problem space is explored. In this case, it is about the specific problem of low metal recycling rates, the causes of which as well as possible options for action can be found at different levels along the entire recycling chain [12] as indicated above. Secondly, the project-based learning method is generated, the design proposal for the ill-defined problem space. It combines the approaches described below from the disciplines of design and psychology. Thirdly, the project-based learning method is evaluated to optimize its alignment with objectives and target group preferences. In the subsequent section the constituent approaches are outlined, culminating in a detailed presentation of the methodical structure, including recommendations for preparation and implementation.

### Project-based learning

Project-based learning is a prevalent method of teaching in industrial design and design engineering, enabling students to acquire and enhance a range of skills through hands-on experience [1]. The active learning process inherent in project-based learning empowers students to engage with real-world problems and develop essential 21st-century competencies, including critical thinking, collaboration, creativity, and problem-solving skills [2,3]. Motivation, as a key component of project-based learning [4,5], is sustained by involving students in meaningful, real-world problems and allowing them to make independent decisions aligned with their individual interests [2,23]. A core principle of project-based learning is to bridge the gap between theory and practice by enabling students to apply their knowledge creatively into practical projects [4]. These projects can produce tangible results, but the primary benefit lies in the actionable insights gained through active participation [5].

### Practical implementation and speculative design task

The action-oriented nature of project-based learning necessitates a clear definition of its goals and the corresponding design process for students to follow. While the production of fully functional and marketable products is not the primary objective, the design task should be conceptual in nature to facilitate a particularly subjective discourse. The corresponding speculative design approach advocated by Dunne and Raby [24] prioritizes the conceptual over the practical, contrasting with project-based learning's focus on problem-solving. This inherent duality should be clearly communicated to students prior to their practical engagement. We're taking a speculative approach on purpose to encourage students to think outside the box, independent of external factors, especially those resulting from economic constraints, becoming a bigger concern in larger projects further on in their studies.

A generative toolkit is provided to guide and support the target group in their practical project work. Rooted in the intersection of design and applied psychology, generative toolkits were initially developed for non-designers to facilitate collaborative design processes and encourage the expression of feelings, ideas, and dreams about future use scenarios [25,26]. The term ‘generative’ emphasizes the forward-looking, exploratory, and constructive nature of this toolkit, reflecting its role in the generative phase of the design process. Toolkits comprise several adaptable components that can be arranged and combined in various ways. Importantly, their design is highly context-dependent [27]. A process of designing generative toolkits at the intersection of speculative design and metal recycling is described by Schoch et al. [28], which was followed in this work.

### Norm activation theory

To clarify the objective of increasing intrinsic motivation more comprehensively, a norm activation model by Matthies [29] for the environmental sector is taken into account when designing the project-based learning method. As in the original version by Schwarz and Howard [30], intrinsic motivation or personal norm, a feeling of moral obligation, serves as a crucial link between problem awareness and subsequent action. Following Matthies [29], the model is employed as a conceptual framework to identify targeted interventions at each cognition of the attention stage, transforming the project-based learning method into a social-psychological intervention. The influence of moral motives on environmentally friendly behavior has been extensively studied and validated in various empirical investigations [31], including the domain of recycling behavior [32].

### Methodical structure

**Fig. 1:**
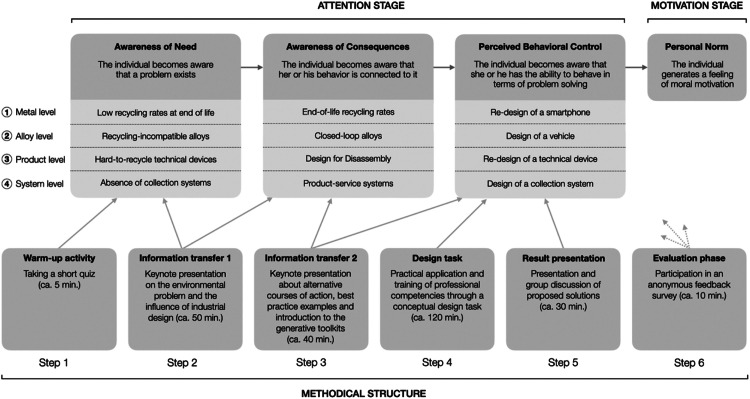
The methodical structure of the project-based learning method and its alignment to the attention stage with its three relevant cognitions as outlined by Matthies [29]. Own depiction.

The designed project-based learning method, as depicted in Fig. 1, is structured around six steps. These steps align with the three cognitions of the attention stage of the norm activation model to activate a feeling of moral obligation. Over the four-day course, the four distinct content levels of i) metal, ii) alloy, iii) product, and iv) system are explored, representing relevant fields for improving metal recycling. Steps two and three focus on promoting specific professional competencies within each of the four levels. These are then practiced in the fourth step and reflected upon during the result presentation. The project-based learning method is framed by an anonymous quiz at the beginning and a feedback survey at the end **(see Supplement A and B for the product level example)**.

The quiz aims to mentally prepare participants for the respective content levels while providing a brief insight into their current knowledge, which can be considered in subsequent discussions. The quiz results are gradually revealed to the entire group in the following second step, allowing for a comparative self-assessment. The final evaluation objectives are detailed in the section *Method validation*. The fifth step's result presentation allows participants to share their findings and consolidate their learning.

The initial kick-off meeting, not depicted in Fig. 1, is crucial for providing participants with the overall context. The amount of information transferred should be tailored to the participants’ academic progress, ideally not exceeding 90 min to allow sufficient time for the first content level. The kick-off is delivered by the instructor as an illustrated keynote presentation.

**Fig. 2:**
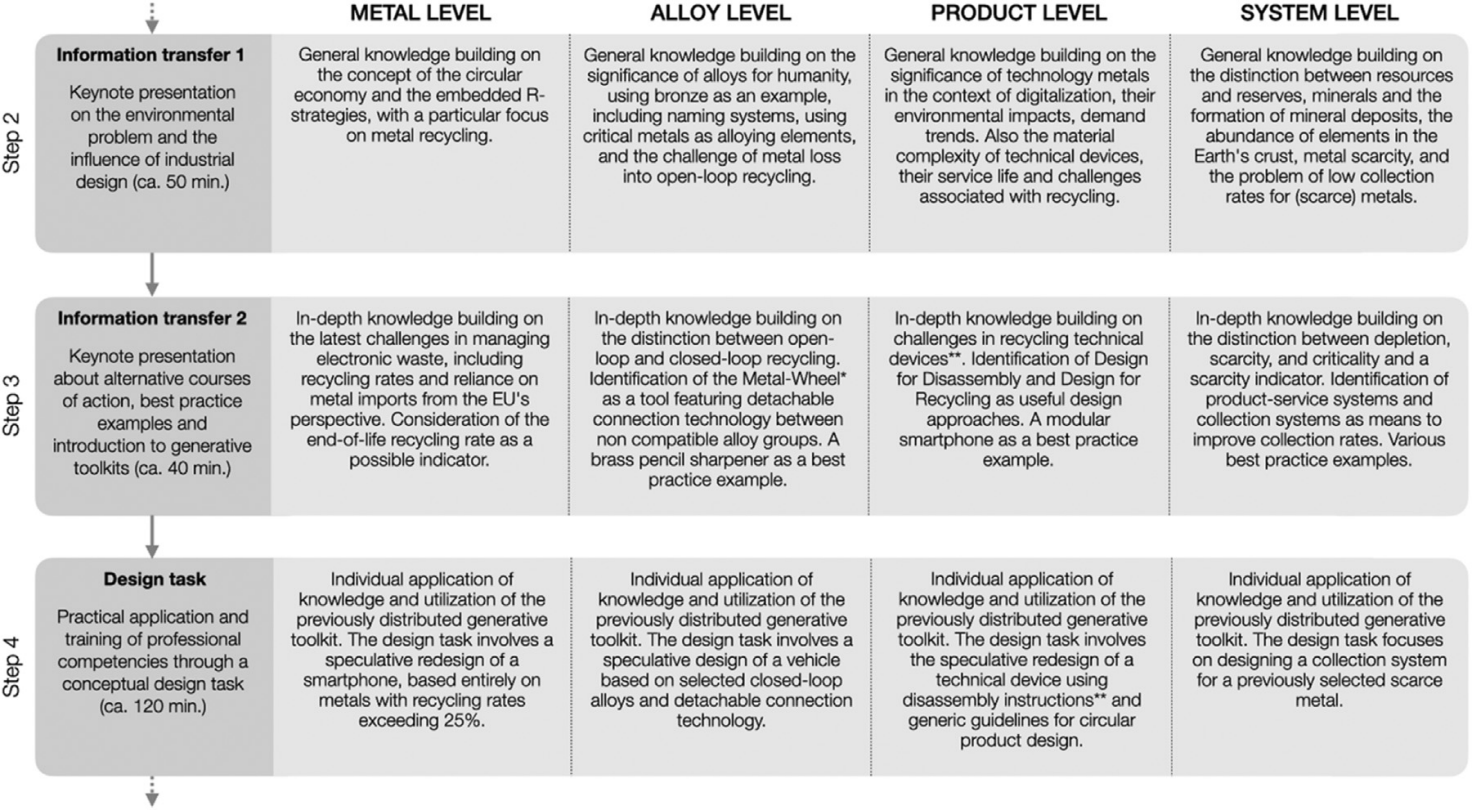
The competence-oriented goals of the four different content levels. *The Metal Wheel is described e.g. in UNEP [15]; **The platform iFixit [33] provides extensive information on how to disassemble electronic devices. Own depiction.

Fig. 2 outlines the specific professional competencies addressed in steps two to four, offering a clearer understanding of the four distinct content levels. The metal level, being the least extensive, is positioned directly after the kick-off to provide a gentle introduction to the project-based learning method for both participants and new instructors. Following a micro-to-macro logic, the four levels build upon each other, offering participants a wide range of opportunities to influence metal recycling through design. However, they can also be addressed independently, as each level has distinct nuances and addresses unique challenges. After the initial information transfer in the second step, each level undergoes a second, more in-depth information transfer in the third step, before design tasks are introduced in the fourth step.

### Generative toolkits: product level example

**Fig. 3:**
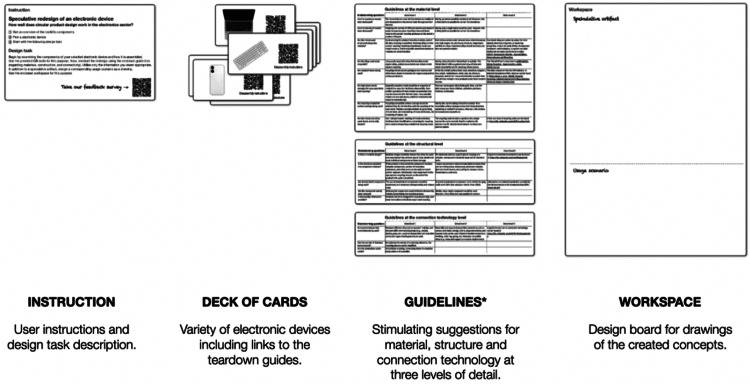
The generative toolkit on the product level consists of four components. *Detailed guidelines for materials, system design and connection technologies are provided below. Own depiction.

**Fig. 4:**
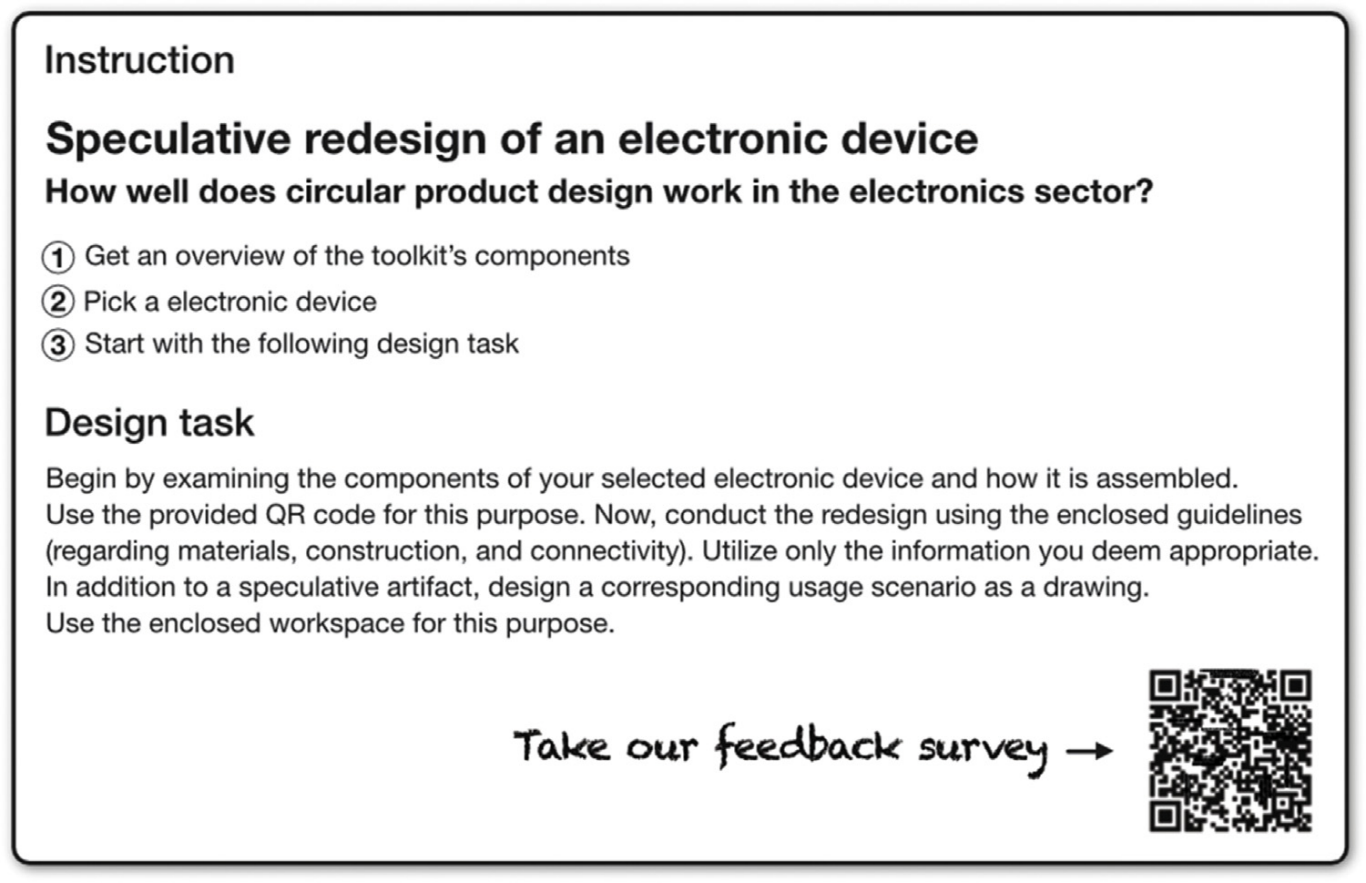
The instructions for all generative toolkits follow a similar format. This is the product level version. Own depiction.

Each content level includes a generative toolkit to support practical application. Fig. 3 provides a compact overview of the product level toolkit, which consists of four main components. The **instruction** reflected in Fig. 4 explains how to use the generative toolkit and how the design task works**.** In addition, a QR code leads to the feedback survey, which is part of the final evaluation (see chapter *Method validation*).

**Fig. 5:**
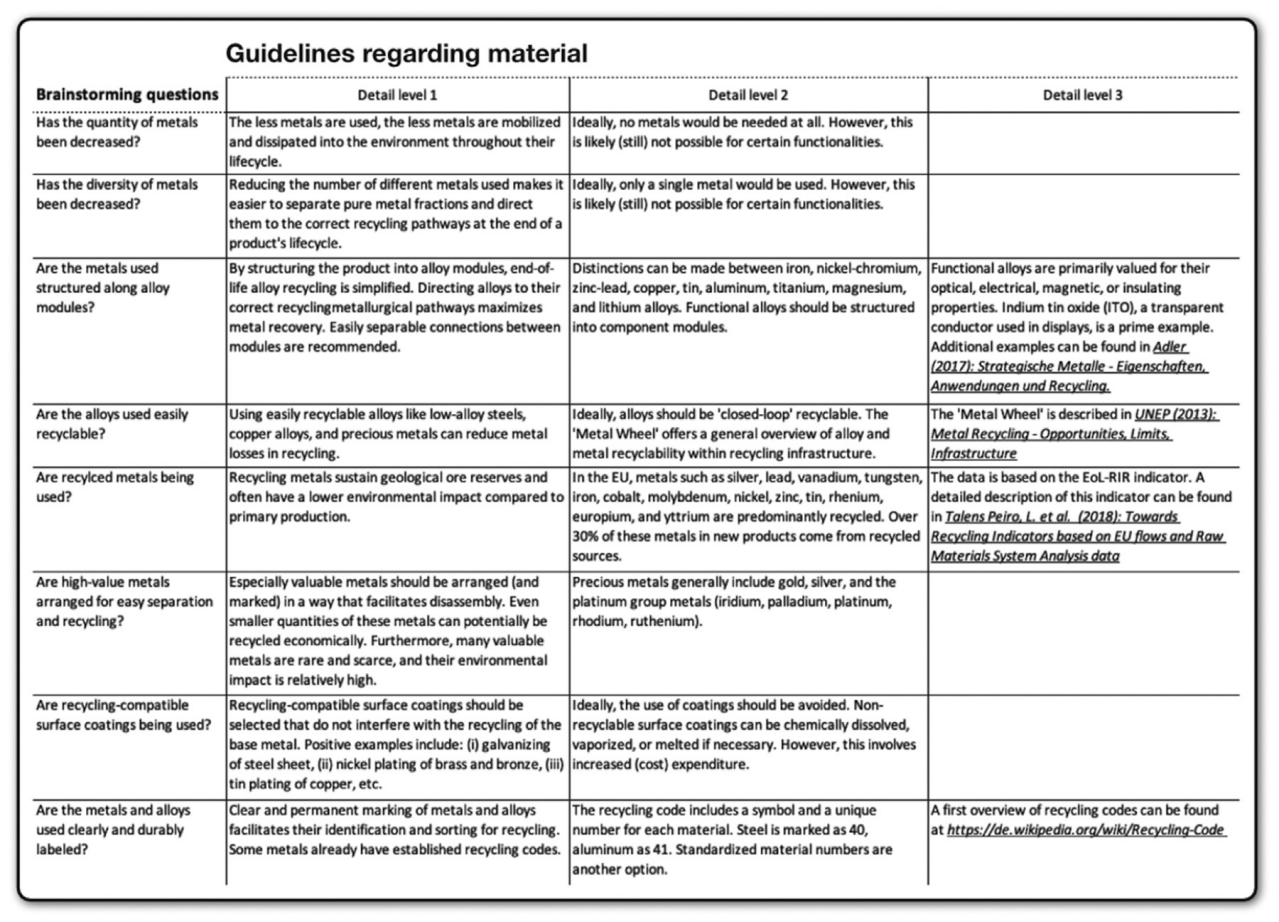
Guidelines regarding material. Own depiction.

**Fig. 6:**
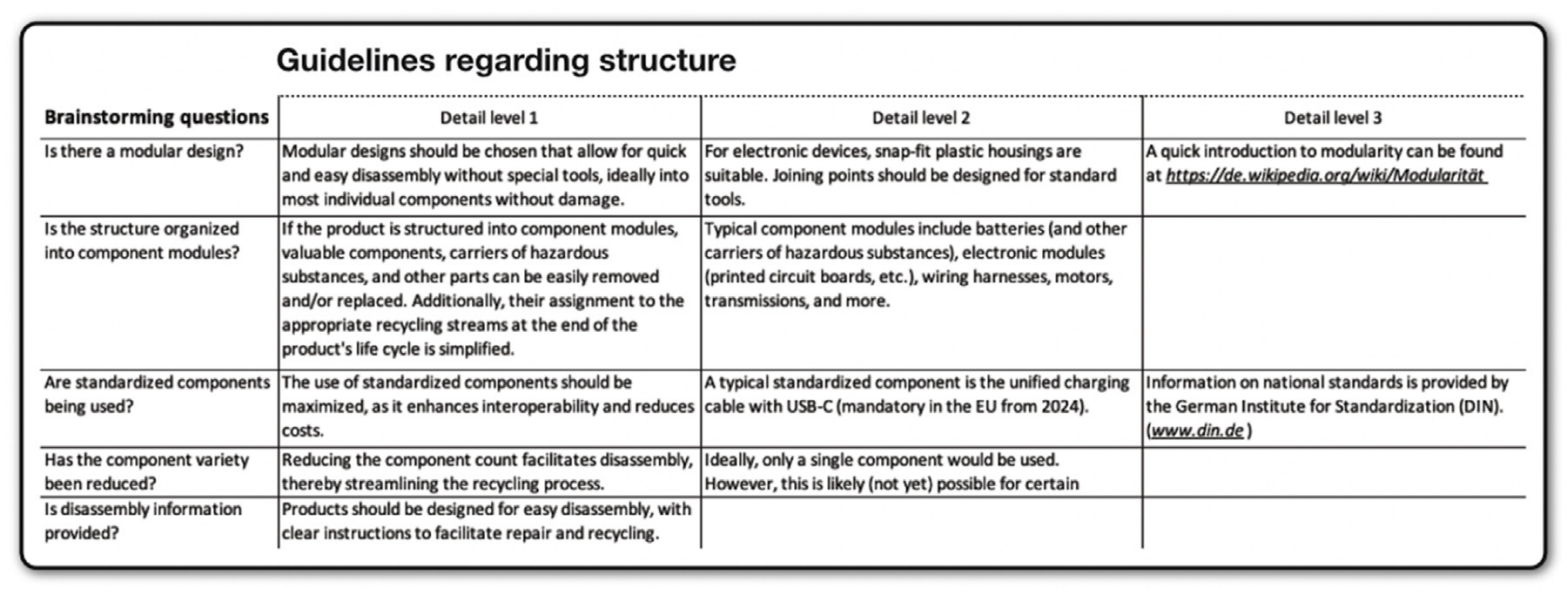
Guidelines regarding structure. Own depiction.

**Fig. 7:**
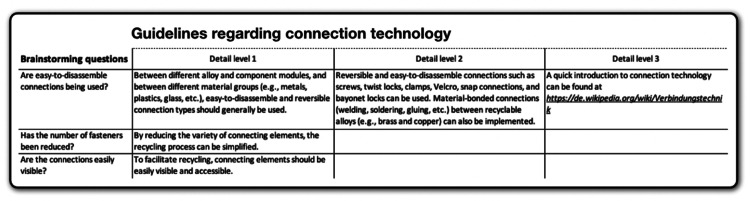
Guidelines regarding connection technology. Own depiction.

The **deck of cards** contains a selection of rather difficult-to-disassemble electronic devices, each linked to the corresponding disassembly guides from iFixit [33], which are freely accessible. Participants will use the **guidelines** reflected in Fig. 5, Fig. 6, Fig. 7 to redesign their chosen electronic device. The guidelines, focusing on metal recycling, aggregate insights from relevant literature, which will not be further elaborated on here due to space constraints. They provide detailed recommendations at the material, structural, and connection technology dimensions. Participants can select any guidelines they deem appropriate for their device and developed concept. It is not intended that all guidelines be considered at once. Whether the concepts created based on the guidelines ultimately result in ecologically, socially, and economically beneficial solutions would have to be examined in detail, but as mentioned above, this is not the subject of the project-based learning method.

**Fig. 8:**
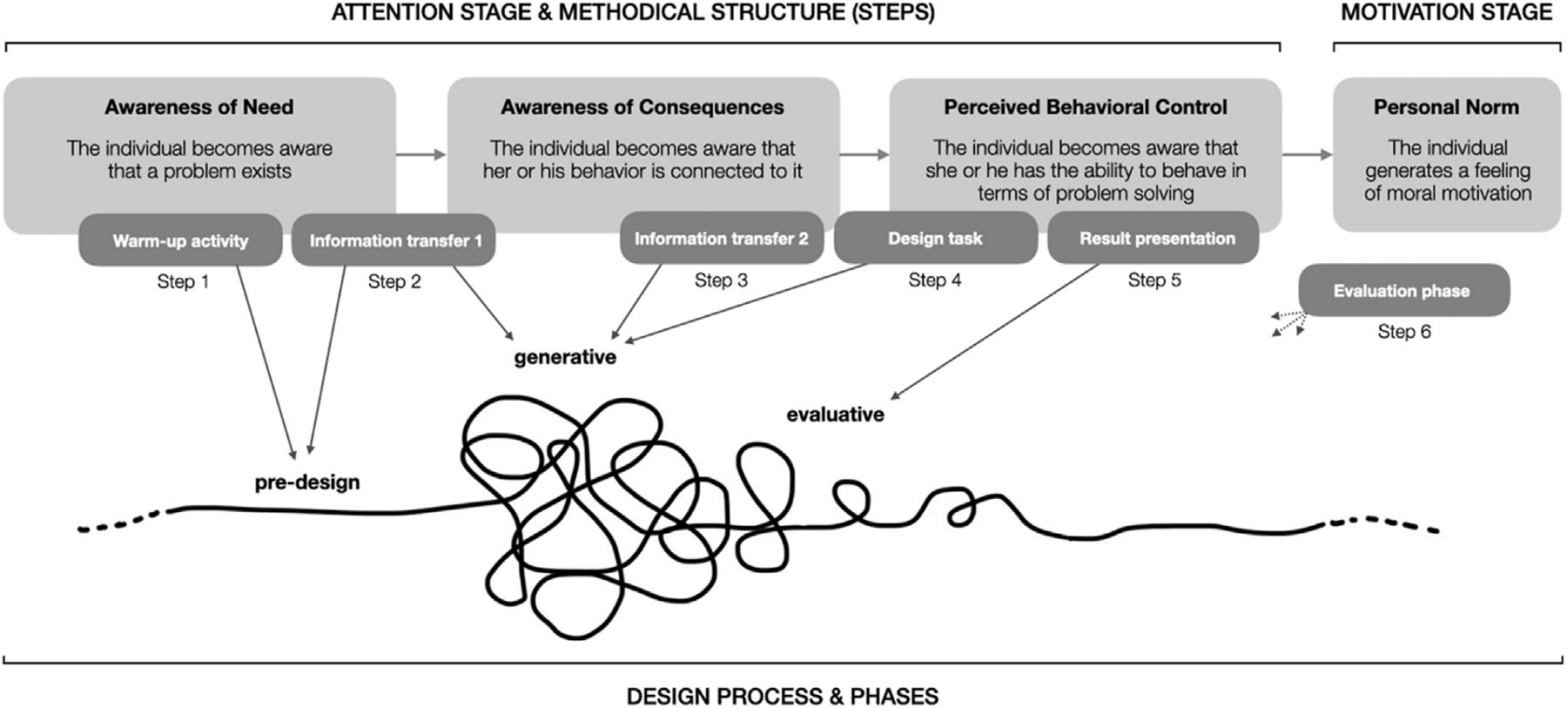
Linkages between the attention stage, the steps of the methodical structure and the relevant phases of a generic design process as outlined by Sanders and Stappers [27]. Own depiction.

In contrast to the connection between the project-based learning method's methodical structure and the attention stage of the norm activation model (Fig. 1), we propose a generic design process (Fig. 8). Our adaption of the model by Sanders and Stappers [27] focuses on the three phases: pre-design, generative and evaluative. The measures integrated into the methodical structure can be described as stimuli to act along these phases. In the generative phase, the fuzzy front-end of a design process, the focus is on generating ideas and concepts. Steps two, three and four guide students’ creative act, steering the design exploration towards the objectives of the project-based learning method. Prior to this, in the pre-design phase via the first two steps, the foundations for participation are laid. Ideally, after the evaluation phase via the fifth step, personal norms are activated.

### Preparation and application

The project-based learning method is guided by a facilitator who should have a basic understanding of the circular economy and metal recycling. Ideally, he or she has a background in industrial design or a similar field to be able to empathize with the participants and adequately address potential needs. The facilitator is responsible for preparing the project-based learning method. This includes setting up the room, which requires a projector for information transfer, as well as preparing the corresponding keynote presentations and printing and cutting out the generative toolkits. Furthermore, the facilitator is responsible for preparing the questionnaires for quizzes and feedback surveys if an evaluation is to be conducted. We used the freely accessible software Google Forms [34] for this purpose. Theoretically, there are no limits to the number of participants as long as there is a suitable room, as the generative toolkits can be produced in any quantity with relatively little effort. The design tasks can be completed individually or in small groups. We recommend the latter. This gives participants the opportunity to interact with each other more intensively, which can have a positive impact on individual self-confidence. It is advisable to conduct steps one to three in the morning until lunchtime to have enough time for the design task in the afternoon. The facilitator should be present, especially at the beginning of the design task, to assist participants with any questions. A short meeting after the first third of the task could be helpful to provide feedback on any uncertainties within the entire group.

## Method validation

Two pilot implementations of the project-based learning method were conducted at Wuppertal University with fourth-semester students in the industrial design program. Novice learners were selected as the target group, as they are likely to have limited prior knowledge or any form of motivation in this context. The module handbook of the study program confirms this general assumption, indicating that the addressed contents of each level are new to the students. While the sample primarily consist of students at the beginning of their careers and who have predominantly grown up in Germany, it is important to note that the proposed project-based learning method, due to its generic design, is essentially culture- and demography-independent and can thus be applied in other contexts. Detailed demographic data was not collected to protect participant privacy. The first trial was conducted over the entire summer semester of 2023, encompassing twelve one-hour course units. Twenty-two students were enrolled in the course. The second trial was a concentrated block course held in May 2024, consisting of four five-hour sessions on four consecutive days. Sixteen students participated.

Although measuring personal norms is considered difficult, it can be approximated by asking participants in questionnaires about their expected feelings [35]. Following this suggestion and as announced during the kick-off, participants completed an anonymous feedback survey after each of the four days of the project-based learning method to evaluate it. Participants access the anonymous feedback survey via a QR code embedded in the generative toolkits, as described above. Additionally, some participants were interviewed to gather more in-depth feedback. Given the primary objective of the overall study to assess the appropriateness of the measures within the methodical structure, the evaluation was predominantly qualitative. However, four measurement points also provided initial insights into the overall effectiveness of the project-based learning method, which are presented here for the second version. The corresponding measurement instrument included both quantitative and qualitative components **(see Supplement B for the product level example).**

**Fig. 9:**
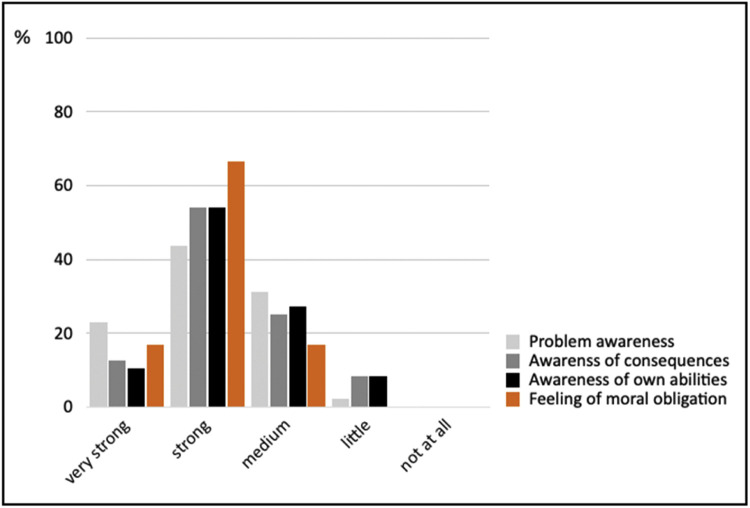
Aggregated indication of the project-based learning method's effectiveness (*n* = 48). Own depiction.

To indicate the suitability of the project-based learning method for increasing intrinsic moral motivation, the results from the written feedback survey are presented in aggregated form across the four parameters: i) problem awareness, ii) awareness of consequences, iii) awareness of own abilities, and iv) feeling of moral obligation (Fig. 9). A total of 48 votes were analyzed for each parameter, distributed across the four content levels. While a detailed analysis of the individual levels was not conducted, the overall results indicate that the personal norm of the target group, their feeling of moral obligation, was strongly activated. Further analysis reveals that problem awareness tended to be addressed more effectively than awareness of consequences and one's own abilities.

## Limitations

We have developed and evaluated a novel project-based learning method to intrinsically motivate industrial design students during their studies to engage in improved metal recycling in their later, longer-term projects. Through the systematic integration of six steps into its methodical structure along the first attention stage of the selected normative decision-making model, we demonstrate which specific intervention measures are potentially suitable in this context. The quantitative part of the evaluation, which is indicated above, suggests that activating moral motivation across students can be successful. However, since it is a relatively small sample size, the insights into effectiveness should only be understood as tendencies. Further research with a larger sample is needed to draw more robust conclusions. It should also be acknowledged that the validity of the self-reported personal norms described by [35] and used for evaluating the project-based learning method depends on several factors, including how well respondents are able to project themselves into hypothetical situations. Another factor, due to the study design, is that statements about long-term effects cannot be made. However, it is likely that the project-based learning method would have to be repeated from time to time, also to reflect new political developments and technological advances in the metal recycling context.

We refer to the normative decision-making model described by Matties [29] and explicitly utilize the first attention stage as a conceptual framework for the project-based learning method design to activate intrinsic motivation among the target group. The overall model shows us that later on, additional social and non-moral motives come into play to determine whether or not a subsequent action actually takes place. This should be kept in mind or could be adapted in future developments. Similarly, the project-based learning method could also be further developed in the future based on more contemporary models that now exist in social psychological research.

## Ethics statements

All authors complied with the MethodsX ethical guidelines.

**If your work involved human subjects,** please include a statement here confirming that the relevant informed consent was obtained from those subjects: The participants agreed that the workshop findings could be used anonymously.

## Declaration of generative AI and AI-assisted technologies in the writing process

During the preparation of this work the author(s) used Google Gemini in order to improve readability. After using this tool/service, the author(s) reviewed and edited the content as needed and take(s) full responsibility for the content of the publication.

## CRediT authorship contribution statement

**K. Schoch:** Methodology, Data curation, Visualization, Validation, Investigation, Writing – original draft. **M. Bickel:** Writing – review & editing. **C. Liedtke:** Supervision, Writing – review & editing. **F. Hemmert:** Supervision, Writing – review & editing.

## Declaration of competing interest

The authors declare that they have no known competing financial interests or personal relationships that could have appeared to influence the work reported in this paper.

## Figures and Tables

**Table:** 

No.	Question
Intro 1.0	As digitalization progresses, so too does the demand for technical or electronic devices and the technology metals they contain. These metals are essential to the digital age but their extraction and processing harm the environment. The carbon footprint, for instance, measures the greenhouse gas emissions associated with producing one kilogram of a particular metal.
Question 1.1	Base metals like magnesium, iron, copper, zinc, and lead have an average carbon footprint of 2.8 kg CO2-eq./kg. Guess: What is the average carbon footprint of technology metals such as tantalum, indium, dysprosium, palladium, and gold? *(Multiple-choice: 420 / 840 / 1680 / 3360)*
Intro 2.0	The smartphone, a common technical device, relies on many technology metals for its various functions. Over 1.5 billion smartphones were sold worldwide in 2022. Service life can be defined as the duration between the first and last use by a single individual.
Question 2.1	Guess: What is the average service life of smartphones in Austria? *(Multiple-choice question: 2.2 years / 2.7 years / 3.2 years / 3.7 years)*
Question 3.0	How strong is your desire to learn more about the topics of technology metals and the service life of products? *(Multiple-choice question: very strong / strong / moderate / weak / not at all)*

**Table:** 

No.	Question
1.0	How strongly did the third part of the course increase your problem awareness of hard to recycle technical devices? *(Multiple-choice question: very strong / strong / medium / little / not at all)*
1.1	Why? (open-ended question)
2.0	How strongly did the third part of the course increase your awareness of how your professional behavior contributes to the problem of hard to recycle technical devices? *(Multiple-choice question: very strong / strong / medium / little / not at all)*
2.1	Why? (open-ended question)
3.0	How strongly did the third part of the course increase your awareness of the existence of alternative courses of action (such as design for disassembly) that can be implemented? *(Multiple-choice question: very strong / strong / medium / little / not at all)*
3.1	Why? (open-ended question)
4.0	How strongly do you feel a moral obligation to actively work towards improving the problem of hard to recycle technical devices in your actual product development work? *(Multiple-choice question: very strong / strong / medium / little / not at all)*
4.1	Why? (open-ended question)

## Supplementary material *and/or* additional information [OPTIONAL]

**A)** Questions for the product level quiz.

**B)** Questions for the product level feedback survey (Excerpt, only questions about the effectiveness for Fig. 9).
